# Bromodomain Proteins in HIV Infection

**DOI:** 10.3390/v5061571

**Published:** 2013-06-21

**Authors:** Daniela Boehm, Ryan J. Conrad, Melanie Ott

**Affiliations:** 1 Gladstone Institute of Virology and Immunology, San Francisco, CA 94158, USA; E-Mails: daniela.boehm@gladstone.ucsf.edu (D.B.); Ryan.Conrad@ucsf.edu (R.J.C.); 2 Department of Medicine, University of California, San Francisco, CA 94158, USA

**Keywords:** bromodomains, HIV, PCAF, PBAF, p300, CBP, TRIM28, BRD2, BRD4

## Abstract

Bromodomains are conserved protein modules of ~110 amino acids that bind acetylated lysine residues in histone and non-histone proteins. Bromodomains are present in many chromatin-associated transcriptional regulators and have been linked to diverse aspects of the HIV life cycle, including transcription and integration. Here, we review the role of bromodomain-containing proteins in HIV infection. We begin with a focus on acetylated viral factors, followed by a discussion of structural and biological studies defining the involvement of bromodomain proteins in the HIV life cycle. We end with an overview of promising new studies of bromodomain inhibitory compounds for the treatment of HIV latency.

## 1. The Bromodomain Protein Family

Reversible modifications of nucleosome components are increasingly acknowledged for their regulatory potential [1]. One such modification is acetylation, which involves the transfer of an acetyl group from acetyl-coenzyme A (acetyl-CoA) to the ε-amino group of lysine side chains on histone and non-histone proteins [2,3]. This modification is catalyzed by lysine acetyltransferases (KATs, also known as HATs), whose action is reversed by lysine deacetylases (KDACS, also known as HDACs) or sirtuins (SIRTs). Histone acetylation is a well-studied modification that antagonizes the nucleosomal DNA-protein interaction, promoting chromatin accessibility and transcriptional activation [4]. However, aside from causing physiochemical changes in the nucleosome core, acetylation also generates novel and unique interaction interfaces for the assembly of macromolecular complexes important for a variety of cellular processes. 

**Figure 1:**
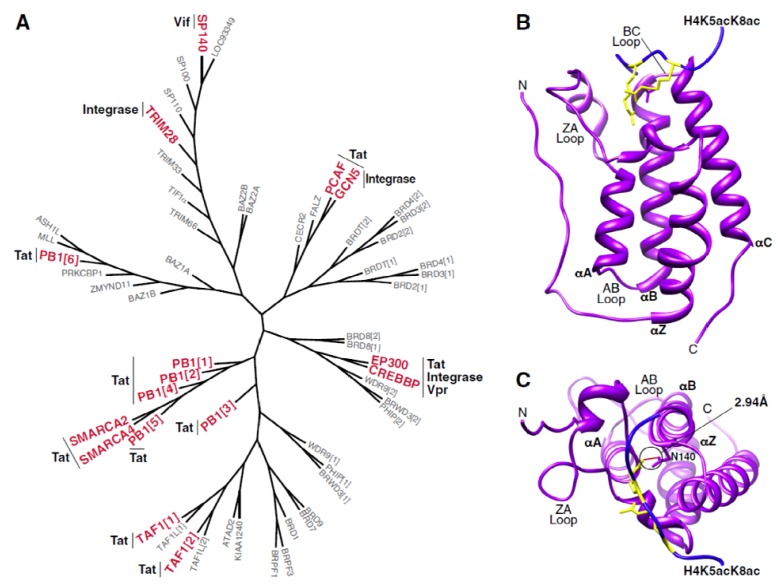
The human bromodomain family. (**A**) Phylogenetic tree of 57 human bromodomains. Those bromodomain-containing proteins that have been shown to interact with HIV proteins are denoted in red with the corresponding viral factor indicated alongside. Phylogenetic trees were generated using Seaview v4.4.1 with individual bromodomain sequences obtained from [23]. (**B**) Structure of the first bromodomain of BRD4 (purple) in complex with a diacetylated histone peptide (blue). Histone acetyllysine residues are shown in yellow. Structural representations in (**B**,**C**) were rendered using Chimera (UCSF) with PDB: 3UVW (**C**) Top view of interaction between first bromodomain of BRD4 and a diacetylated histone peptide. The hydrogen bond between the canonical bromodomain asparagine residue (N140 in BRD4) and the histone acetyllysine residue is shown in red with an estimated length of 2.94A.

The bromodomain is a conserved protein module of ~110 amino acids that recognizes and binds ε-*N*-acetylated lysine residues in histone and non-histone proteins [5,6]. Recognition of acetyl-lysine residues by bromodomain-containing proteins is at least partly responsible for the functional consequences linked to protein acetylation. The first reference to a bromodomain can be traced to the Drosophila gene *brahma* (*brm*) [7], and the human bromodomain family to date includes 46 distinct proteins and 61 unique bromodomains (Figure 1A) [8]. Select transcriptional regulators (*i.e.*, BRD4, TAF1, TIF1), chromatin-modifying enzymes (*i.e*., p300, PCAF, MLL), and nucleosome remodelers (*i.e*., SMARAC2, PB1, BAZ1B) contain bromodomains [6]. 

Structurally, bromodomains are comprised of four left-handed α-helices (αZ, αA, αB, and αC) connected by two loops (ZA and BC loops) (Figure 1B) [5]. This structure forms a deep hydrophobic cavity that serves as the acetyl-lysine recognition site [8]. While the helical regions are moderately conserved among different bromodomains, the loop regions are highly variable in both length and sequence composition [8]. This loop variability guides the substrate specificity observed among members of the bromodomain family. Co-crystal structures of bromodomains with various acetylated peptides demonstrate that the neutralized acetyl-lysine residue forms a hydrogen bond with an asparagine residue found in most bromodomains (Figure 1C). Some bromodomains exhibit higher affinity for multiply acetylated substrates, while the affinities of others are regulated by additional post-translational modifications of the ligand, such as phosphorylation [8,9].

## 2. HIV Infection and Reversible Acetylation

Reversible acetylation of histone and non-histone proteins plays a key role in HIV transcription and is a lead target in preclinical and clinical efforts to reverse HIV latency [10,11,12,13,14]. Upon integration into the human genome, the HIV proviral cDNA is organized into higher-order chromatin and becomes subject to regulation by host chromatin-modifying enzymes, including acetyltransferases and deacetylases [15]. Indeed, it has been shown that following stimulation with phorbol esters, distinct lysines in histone H3 (H3K9 and H3K14) become rapidly acetylated within a single nucleosome (nuc-1) located immediately downstream of the viral transcription start site [16]. However, the HIV provirus differs from cellular genes because it encodes a viral protein called transactivator of transcription (Tat). Tat is essential for HIV replication. It relieves a powerful block to the elongation of HIV transcripts by cooperatively binding to (1) an RNA stem-loop structure called TAR present at the 5' end of all nascent viral transcripts and (2) the positive transcription elongation factor b (P-TEFb), which together with other elongation factors, including PAF1c, forms a “super-elongation complex” [17,18].

Additionally, Tat is known to recruit several acetyltransferases to the HIV LTR, thus enhancing HIV transcription in the context of chromatin. These include KAT3B/EP300/p300 [19], KAT3A/CREBBP/CBP [19], KAT2A/GCN5 [16], and KAT2B/PCAF [16]. Conversely, several HDACs have been shown to bind to the HIV promoter located in the 5' long terminal repeat (LTR) through interactions with cellular transcription factors, including YY1 [20], LSF [20], NF-κB [21], AP-4 [22], CBF-1 [23], c-Myc and Sp1 [24]. HDAC inhibitors, which are known to activate HIV from latency in cell culture models [25], are being clinically tested for their potential to reactivate HIV from transcriptional latency [10]. 

Tat itself is subject to reversible acetylation. Tat is acetylated by KAT2B/PCAF at lysine 28 within a characteristic cysteine-rich region required for its interaction with P-TEFb [26]. Tat is also acetylated by KAT3B/EP300/p300 and the close KAT2B/PCAF homologue KAT2A/GCN5 at lysines 50 and 51 located in its basic RNA-binding domain [26,27,28]. Both acetylation events positively support Tat’s transcriptional activity [29,30] and are reversed by the deacetylase activities of HDAC6 and SIRT1 [31,32]. In addition to Tat, HIV integrase, a DNA-binding protein that catalyzes 3' processing and strand transfer of the viral genome, is acetylated by KAT3B/EP300/p300 at lysines 264, 266 and 273 [33]. These residues are also subject to acetylation by KAT2A/GCN5, in addition to lysine 258 [34]. Integrase acetylation increases the affinity of the enzyme for DNA and also enhances strand-transfer catalysis *in vitro*. Lastly, Vpr, a viral protein implicated in nuclear translocation of the HIV pre-integration complex, HIV-mediated G_2_/M arrest, and transcription of viral and cellular promoters, interacts with KAT3B/EP300/p300 [35]. Mutational analysis suggests that the Vpr-p300 interaction occurs independently of the p300 bromodomain [35], and it is unclear whether Vpr is acetylated.

## 3. Interactions between Bromodomain-Containing Proteins and HIV Proteins

### 3.1. Acetylation-Dependent Interactions

#### 3.1.1. p300/CBP-Associated Factor (PCAF)

**Figure 2:**
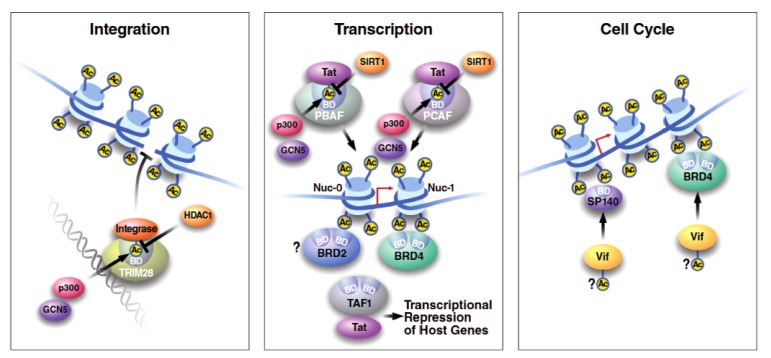
Role of bromodomain proteins in HIV infection. Bromodomain proteins implicated in HIV transcription, HIV integration, and cell cycle progression are schematized. Briefly, acetylated integrase displays enhanced enzymatic activity, yet generates an interaction interface for the TRIM28 bromodomain that in turn recruits the HDAC1 deacetylase, negatively impacting HIV integration. With respect to viral transcription, Tat acetylated at K50/51 interacts with BAF180 and Brg-1 within the PBAF complex to support viral transcription. Acetylated Tat also interacts with p300/CBP-Associated Factor (PCAF) to induce local acetylation of histones and potentially other factors at the site of viral transcription. BRD4 is present at the HIV long terminal repeat (LTR), yet is hypothesized as an intracellular competitor of Tat, while the role of BRD2 in HIV transcription is unknown. Tat also interacts with TAF1 to repress select cellular promoters. BRD4 and SP140 are both cell cycle regulators that interact with Vif, yet the bromodomain-dependence and functional significance of these interactions remain unclear.

KAT2B/PCAF (p300/CBP-associated factor) is a histone acetyltransferase that contains a *N*-acetyltransferase domain and a *C*-terminal bromodomain. KAT2B/PCAF also participates in the reversible acetylation of various non-histone proteins, including p53, β-catenin, retinoblastoma protein (Rb), and several transcriptional regulators, such as the general transcription factors TF_II_Eβ and TF_II_F and the sequence-specific transcription factors E2F1, c-Myc, and MyoD (reviewed in [36]) [37]. In HIV infection, KAT2B/PCAF acetylates Tat at lysine 28 [26,38]. Acetylation of Tat on lysine 28 facilitates recruitment of P-TEFb kinase, resulting in the efficient phosphorylation of the heptad repeats in the carboxyl terminus of cellular RNA polymerase II and thus promoting HIV transcript elongation [26,29,38]. KAT2B/PCAF is recruited to Tat through an acetylation-dependent mechanism (Figure 2). Acetylated K50 in Tat acts as a specific binding partner of the KAT2B/PCAF bromodomain, an interaction that was examined by NMR spectroscopy at the structural level and characteristically involves additional residues in Tat that interact with the KAT2B/PCAF bromodomain in an acetylated K50-dependent manner [39,40]. KAT2B/PCAF binding to Tat positively supports HIV transcription either through enhanced lysine 28 acetylation in Tat, or enhanced local histone acetylation during HIV transcriptional elongation. Consequently, mutations in the KAT2B/PCAF bromodomain that suppress interactions with acetylated Tat or treatment with small molecules that specifically bind the KAT2B/PCAF bromodomain effectively suppress Tat transactivation, supporting the concept that this interaction could serve as a specific target for anti-HIV transcription therapeutics [39,41]. 

#### 3.1.2. SWItch/Sucrose Non-Fermentable (SWI/SNF)

Members of the SWI/SNF family form evolutionarily conserved chromatin-remodeling complexes that utilize the energy of ATP hydrolysis to induce nucleosome remodeling. Two important and distinct SWI/SNF complexes have been described in humans: BAF and PBAF. These complexes contain either Brg-1 (also known at SMARCA4) or BRM (also known at SMARCA2) as the major catalytic subunit together with several accessory proteins. BAF is distinguished by the presence of the BAF250a/b subunit, while PBAF contains BAF180 (also known as Polybromo or PB1), BAF200, and BRD7 subunits. BAF has been shown to repress HIV transcription by positioning the nuc-1 nucleosome immediately downstream of the transcription start site in a manner that encumbers processive transcription [42]. Such repressive remodeling events may be crucial for the maintenance of HIV latency in the absence of Tat. Conversely, the PBAF complex is required for robust Tat-mediated transactivation of HIV expression [43,44]. Both shared constituents of BAF and PBAF, Brg-1 and BRM, interact with Tat. These interactions are regulated by acetylation of Tat, with K50 acetylation enhancing the Tat-Brg1 interaction [45] and inhibiting the Tat-BRM interaction [46]. The interaction between acetylated Tat and Brg-1 was mapped to the Brg-1 bromodomain, while the Tat-BRM interaction was shown to be bromodomain-independent [46]. Tat displays enhanced interaction with the PBAF-specific constituent BAF200 in a K50/K51-dependent manner [47]. In addition, Tat acetylated at K50/K51 interacts with PBAF through the BAF180 subunit, an interaction that facilitates Tat-mediated transactivation (Figure 2) [42]. It is likely, though not experimentally confirmed, that acetylated Tat interacts with one or more of the six bromodomains present in BAF180. These findings evoke a model in which Tat acetylation at K50/K51 functions to switch the repressive BAF complex with the activating PBAF complex through specific interactions with several bromodomain-containing proteins in the PBAF complex, including Brg-1 and BAF180. 

#### 3.1.3. Bromodomain-Containing Protein 4 (BRD4)

BRD4 is a mitotic chromosome-associated protein that serves as an important regulator of post-mitotic transcription by recruiting various transcriptional regulators to acetylated chromatin. BRD4 is also required for maintaining a proper higher-order chromatin structure [48,49,50]. BRD4 is a member of the bromodomain and extraterminal domain (BET) family of bromodomain proteins. Members of the BET family are distinguished by the presence of two functional domains—tandem bromodomains and a so-called extraterminal domain, the latter of which may serve to mediate protein-protein interactions [49]. BRD4 contains a third functional domain termed the P-TEFb-interacting domain (PID) [51]. The PID serves to recruit and activate the Tat cofactor P-TEFb, a heterodimer composed of cyclin T1 and CDK9 that when complexed with the HEXIM1 inhibitor is part of an inactive ribonucleoprotein complex found in HeLa cells and other tumor cell lines [52,53]. While BRD4 is an important factor recruiting P-TEFb to the HIV promoter in the absence of Tat, Tat and the BRD4 PID compete for P-TEFb binding, making BRD4 a negative factor in Tat transactivation [51,54]. In addition, BRD4 negatively regulates HIV transcription by inducing an inhibitory phosphorylation event on CDK9 [55].

The cyclin T1 subunit of P-TEFb is acetylated at four defined residues by KAT3B/p300 [56]. Three of the four acetylated residues (K380, K386, K390) bind the second bromodomain of BRD4 defining a second acetylation-dependent P-TEFb interaction site in BRD4 besides the PID. Interestingly, while this acetylation-dependent interaction is required for basal HIV LTR activity and cellular gene expression, it is not necessary for Tat-mediated transactivation of HIV transcription, supporting the model that Tat- and BRD4-mediated activities at the HIV promoter are mutually exclusive [51,54]. Vollmuth *et al.* determined the crystal structures of the two bromodomains of BRD4 and showed that acetylated K390 weakly bound to the second bromodomain of BRD4 [57]. This binding is markedly enhanced when K380 and K386 are also acetylated [52], supporting an emerging model that bromodomains of BET proteins have a preference for binding to multiply acetylated proteins. 

#### 3.1.4. Tripartite Motif-Containing Protein 28 (TRIM28)

TRIM28 was originally identified as an interaction partner of members of the family of Krüppel-associated box (KRAB) domain-containing zinc finger transcription factors. It is also named KRAB-associated protein 1 (KAP1), KRAB-A-interacting protein 1 (KRIP1), and transcription intermediary factor (TIF) 1β. Its protein architecture includes an *N*-terminal tripartite motif (TRIM) that acts as a protein-protein and oligomerization interface, and contains a RBCC (Ring finger, two B-box zinc fingers, and a coiled coil) domain, a central heterochromatin protein 1 (HP1)-binding domain, a TIF1 signature sequence (TSS) domain, and a *C*-terminal plant homeodomain (PHD) and bromodomain. The TRIM28 bromodomain is a 100-amino acid stretch consisting of four α-helices that, similar to other bromodomain-containing proteins, has a conserved hydrophobic core and recognizes the backbone of histone tails [58]. Structural analysis of the tandem PHD and bromodomain (PB) of TRIM28 between amino acids 624 and 812 revealed that both domains function as a cooperative unit to facilitate lysine sumoylation, which is required for TRIM28 co-repressor activity in gene silencing [58]. In a yeast two-hybrid screen, TRIM28 was identified as an interaction partner of acetylated HIV-1 integrase [59]. TRIM28 *in vitro* and *in vivo* preferentially binds integrase when acetylated at K264, K266 and K273, rather than the unmodified protein, implicating that this interaction is mediated by the TRIM28 bromodomain. Integrase K264, K266 and K273 are targeted for acetylation by KAT3B/p300, which is a prerequisite for the interaction of TRIM28 with integrase and leads to subsequent recruitment of HDAC1. As a consequence, TRIM28 inhibits HIV-1 integration through integrase deacetylation by HDAC1 (Figure 2) [60]. 

### 3.2. Other Interactions with Bromodomain-Containing Proteins

#### 3.2.1. Transcription Initiation Factor TFIID, Subunit 1 (TAF1)

TAF1, also referred to as TAF_II_250, is the largest component of transcription factor TFIID that is composed of TATA-binding protein (TBP) and a variety of TBP-associated factors. TAF1 contains *N*- and *C*-terminal serine/threonine kinase domains, but can also function as an acetyltransferase and an ubiquitin-activating/conjugating enzyme. It contains two tandem bromodomains, both located in the *C*-terminus of the protein. Structurally, the two bromodomains form two side-by-side, four-helix bundles, each with an acetyl lysine binding pocket at its center, recognized by diacetylated histone H4 peptides [61]. When Weissman *et al.* identified TAF1 as a Tat interaction partner, the interaction was mapped to amino acids 80 to 83 in Tat and a site in TAF1 that overlaps the acetyltransferase domain, inhibiting TAF1 acetyltransferase activity [62]. TAF1 was not required for Tat transactivation of the HIV LTR; instead, the interaction with TAF1 was linked to Tat-mediated transcriptional repression, *i.e*., of the MHC class I promoter (Figure 2) [62]. These findings could be explained by a model in which the interaction with Tat inhibits TAF1 acetyltransferase activity, thereby recruiting an inactive Tat/TAF1 complex to actively transcribing gene promoters containing hyperacetylated histone H4. 

#### 3.2.2. Nuclear Body Protein SP140

SP140, also referred to as lymphoid-restricted homolog of Sp100 (LYSp100) is a component of a subset of nuclear bodies in lymphoid cells. It is involved in the pathogenesis of acute promyelocytic leukemia and has been shown to be an autoantigen in primary biliary cirrhosis [63,64,65]. Its protein architecture includes a *N*-terminal homogeneous staining region (HSR) domain, a central SAND domain which mediates DNA binding, a PHD-type zinc finger, and a *C*-terminal bromodomain. In a yeast two-hybrid screen, SP140 was identified as an interaction partner of the HIV Vif protein [66]. Vif enhances the infectivity of HIV virions released from so-called non-permissive cells by inducing the degradation of an antiviral restriction factor APOBEC3G [67]. SP140 was found specifically in non-permissive cells, and HIV-1 infection induced its dispersal from nuclear bodies into cytosolic colocalization with Vif [66]. SP140 interacts with the N-terminal and central regions of Vif (amino acids 1–112) in the yeast two-hybrid screen, and the SP140 prey cDNAs that were isolated encoded the *C*-terminal region between amino acids 527 and 836, which includes the SAND domain, the PHD-type zinc finger, and the bromodomain [66]. Further studies are needed to determine whether an acetyl-lysine-bromodomain interaction is involved in the interaction between Vif and SP140. 

#### 3.2.3. BRD2

BRD2, formerly named RING3 (really interesting new gene 3) or Fshrg1 (female sterile homeotic related gene 1), is a nuclear serine/threonine kinase possessing chromatin binding and transcription activity. Along with BRD4, BRD2 is a member of the BET family, but lacks a *C*-terminal PID domain. 

However, BRD2 was found to coimmunoprecipitate with CDK9/Cyclin T1 or Cyclin T2 [68]. BRD2 also functions as a Tat-independent suppressor of HIV transcription in latent cells [69]. In cell lines containing latent HIV, lentiviral shRNA-mediated depletion of BRD2 resulted in activation of the HIV LTR, and this effect was independent of Tat. The fact that BRD2 is known to bind co-repressor complexes including HDACs [70] supports a model whereby BRD2, by recruiting repressor complexes to the latent HIV LTR, directly suppresses HIV transcription. It remains to be seen whether BRD2, like BRD4, interacts with P-TEFb in an acetylation-dependent manner. 

#### 3.2.4. BRD4

In addition to its role in HIV transcription, BRD4 has been implicated in Vif-mediated cell-cycle progression. By mass spectrometry, Wang *et al.* identified BRD4 and CDK9 as Vif interactors required for Vif-mediated acceleration of cell-cycle transition from the G_1_-to-S phase [71]. BRD4 also regulates the G_2_-to-M transition and stimulates cell-cycle progression from G_1_ to S through recruitment of P-TEFb to chromosomes and stimulation of G_1_ gene expression during late mitosis [72,73]. It is unknown whether the Vif-BRD4 interaction involves the BRD4 bromodomains. 

## 4. Bromodomain Inhibitors and HIV Infection

The characteristic architecture of the bromodomain-acetyl-lysine interface represents a potential target for the development of small-molecule inhibitors. In initial attempts to identify bromodomain inhibitors, NMR-based screens of commercial compound libraries were used to identify compounds that inhibit Tat transactivation at the Tat-PCAF interface [41]. Two lead compounds were discovered using this approach, both with relatively low IC_50_ values for the Tat-PCAF interaction *in vitro*. Recently, several high-affinity binding molecules for bromodomains of the BET family were described [74,75,76,77,78]. JQ1, a thienodiazepine derived from a BRD4 ligand developed by Mitsubishi Pharmaceuticals [79], was shown to bind the first bromodomain of BRD4 with high affinity and target the second bromodomain of BRD4, and those of BRD2, BRD3, and BRDT [75]. MS417, a BET inhibitor derived from JQ1, specifically targets the interaction between BRD4 and the acetylated p65/RelA subunit of the transcription factor NF-kB and has potent anti-inflammatory effects in a mouse model of HIV-associated kidney disease [78]. I-BET, a synthetic “histone mimic” identified using an ApoA1 reporter system, also functions as a potent anti-inflammatory agent that suppresses expression of pro-inflammatory genes in activated macrophages and confers protection against lipopolysaccharide-induced endotoxic shock and bacteria-induced sepsis [76]. These and other compounds are excellent tools to study BET protein function, but have also shown impressive preclinical promise for the treatment of the NUT midline carcinoma (JQ1, [75]), specific types of leukemia (JQ1 [80], I-BET151 [77]) inflammation (I-BET [76], MS417 [78]), and viral infections, including HIV.

Current antiretroviral therapy only suppresses viral replication, requiring life-long adherence to continuously limit viral loads, but does not eradicate virus from most infected people. Therefore, novel treatments that eliminate persistent viral reservoirs and thereby cure patients are needed. One approach is to reactivate proviral genomes in latently infected cells in order to “purge” viral reservoirs, either through the immune system or through the cytopathic effects associated with viral reactivation. HDAC inhibitors, including valproic acid and vorinostat (SAHA), show great promise as anti-latency therapeutics and have already begun to be tested in clinical trials [10,11,81,82]. Recently, a flood of publications demonstrated that BET bromodomain inhibitors reactivate HIV from latency in cell lines and primary T-cell models (summarized in Table 1). Notably, cells treated with drugs like JQ1 show little synergy with vorinostat, indicating that both drugs target similar pathways in HIV reactivation. However, it is unclear whether any of the current *ex vivo* models faithfully recapitulates the *in vivo* situation of latently infected cells; further studies are needed to evaluate the clinical potential of BET inhibitors in primary T cells. 

**Table 1:** Selected reported bromodomain inhibitors tested in models of HIV latency.

Compound	Model of HIV latency	Effect	Reference
JQ1	Ach2	reactivation	[83]
JQ1	U1	reactivation	
JQ1	J-Lat 10.6	reactivation	
JQ1	Acutely infected primary CD4^+^ cells	reactivation	
JQ1	J∆K	reactivation	[84]
JQ1	J-Lat A2	reactivation	[85]
JQ1	Jurkat 1G5	reactivation	
JQ1	HeLa NH1 and NH2	reactivation	
JQ1	HeLa-T4	reactivation	[86]
JQ1	Primary CD4^+^ T cells	reactivation	
JQ1	Primary CD4^+^ T cells	inhibition	
JQ1 + Prostratin or PMA	J-Lat 6.3	reactivation	
JQ1 + Prostratin or PMA	J-Lat 8.4	reactivation	
JQ1 + Prostratin or PMA	J-Lat 9.2	reactivation	
JQ1 + Prostratin or PMA	J-Lat 15.4	reactivation	
JQ1	J-Lat A2	reactivation	[87]
JQ1	J-Lat A72	reactivation	
JQ1	infected primary Bc12-transduced CD4^+^ T cells	reactivation	
JQ1	infected primary nonpolarized T helper cells	no reactivation	
I-BET	infected primary Bc12-transduced CD4^+^ T cells	reactivation	
I-BET	infected primary nonpolarized T helper cells	no reactivation	
I-Bet151	J-Lat A2	reactivation	
I-Bet151	J-Lat A72	reactivation	
I-Bet151	infected primary Bc12-transduced CD4^+^ T cells	reactivation	
I-Bet151	infected primary nonpolarized T helper cells	no reactivation	
MS417	J-Lat A2	reactivation	
MS417	J-Lat A72	reactivation	
MS417	infected primary Bc12-transduced CD4^+^ T cells	reactivation	
MS417	infected primary nonpolarized T helper cells	no reactivation	

## 5. Concluding Remarks

The acetyl-lysine-bromodomain interface, first therapeutically explored in HIV infection, represents an important regulatory axis that controls many aspects of HIV infection, including viral integration, Tat transactivation, HIV latency, cell-cycle progression of infected host cells, and virally induced inflammation. Furthermore, it is likely that new molecular functions for bromodomain-containing proteins in HIV infection await discovery. We expect that the use of existing and the development of novel bromodomain inhibitors will facilitate both the study and the treatment of HIV infection. 
